# The interplay of dietary sugar, chronic inflammation, and bladder cancer: mechanistic insights, evidence, and prevention strategies

**DOI:** 10.3389/fimmu.2026.1731784

**Published:** 2026-02-09

**Authors:** Larisa Tratnjek, Aleksandar Janev, Tadeja Kuret, Urška Dragin Jerman

**Affiliations:** Institute of Cell Biology, Faculty of Medicine, University of Ljubljana, Ljubljana, Slovenia

**Keywords:** bladder cancer, chronic inflammation, dietary sugar, hyperglycaemia, lifestyle strategies, therapeutic approaches

## Abstract

High dietary sugar intake has emerged as a key modulator of systemic inflammation and metabolic dysregulation, both of which are associated with an increased risk of several chronic diseases, including cancer. Although bladder cancer is primarily driven by factors such as smoking and occupational exposures, metabolic dysregulation may also play a contributory role. Experimental studies indicate that elevated glucose levels promote proliferation, epithelial-mesenchymal transition, increase invasion, and reduce autophagy in bladder cancer cells. Epidemiological evidence suggests associations of high dietary glycaemic index/load and high sugar consumption with bladder cancer risk, although findings for these dietary factors remain heterogeneous. Furthermore, epidemiological data consistently demonstrate a positive association between diabetes mellitus and increased bladder cancer incidence and adverse clinical outcomes. Mechanistically, hyperglycaemia and accumulation of advanced glycation end products (AGEs) can activate inflammatory signalling pathways, including NF-κB, MAPK, and the NLRP3 inflammasome, leading to increased cytokine production, immune dysregulation, and oxidative stress. High dietary sugar intake has also been shown to alter gut microbiota composition, typically reducing short-chain fatty acid (SCFA)-producing bacteria and promoting intestinal permeability, endotoxaemia, and sustained immune activation through TLR4-dependent pathways. Within the bladder tumour microenvironment, systemic inflammatory disturbances enhance oncogenic signalling cascades such as COX-2, JAK/STAT3, and NF-κB, thereby fostering epithelial-mesenchymal transition, angiogenesis, and potential resistance to therapy. Evidence suggests that maintaining well-regulated blood sugar levels may help lower the risk of bladder cancer. Adopting lifestyle habits such as whole-food, fibre-rich diets, probiotics, and regular physical activity supports metabolic and microbial homeostasis, SCFA-mediated immune regulation, and inflammation reduction, thereby serving as a preventive strategy. This review aims to synthesise current evidence on the complex interplay between dietary sugar intake, gut microbiota dysregulation, systemic inflammation, and bladder cancer, and to highlight potential preventive dietary interventions.

## 1 Introduction

Bladder cancer is one of the most common cancers worldwide, with the highest incidence in developed countries (1). Several factors contribute to its development, including genetic predisposition, exposure to carcinogens such as cigarette smoke and industrial chemicals used in the manufacture of dyes, rubber, or paints, as well as infection with *Schistosoma haematobium* and chronic bladder irritation (2, 3). Age and gender also play an important role in the onset and progression of the disease (4). Bladder cancer is a major global health challenge, currently ranking as the ninth most diagnosed cancer worldwide. According to GLOBOCAN 2022, there were an estimated 613,791 new cases, 220,349 deaths, and a five-year prevalence of approximately 1.95 million patients globally (5). In addition to its substantial human toll, bladder cancer imposes a significant economic burden, with one of the highest lifetime treatment costs per patient among all cancers due to high recurrence rates and intensive surveillance requirements (6). Bladder cancer exhibits marked disease heterogeneity, with most cases presenting as non-muscle-invasive bladder cancer (NMIBC), a form that by definition does not invade the detrusor muscle (7), while a smaller but clinically significant proportion progress to muscle-invasive disease (MIBC) (8). NMIBC is defined by superficial papillary tumours that rarely invade the detrusor muscle but show high recurrence rates. In contrast, MIBC is an aggressive, genomically unstable subtype characterized by deep invasion into the bladder wall and a markedly poorer prognosis (9). There is increasing evidence that, in addition to genetic and environmental influences, metabolic and nutritional factors also play a role in the development of the disease. In particular, elevated blood glucose levels known as hyperglycaemia, insulin resistance, and diabetes mellitus (DM), have been linked to an increased risk of bladder cancer and poorer clinical outcomes (10, 11). While both NMIBCs and MIBCs generally rely on enhanced glycolysis to sustain cell proliferation, MIBC may also exhibit metabolic plasticity (hybrid glycolysis/oxidative phosphorylation phenotype) that could facilitate adaptation to metabolic stress (12). Elevated fasting plasma glucose (FPG) was estimated to account for approximately 22,823 deaths and 399,655 disability-adjusted life years (DALYs) globally for bladder cancer in 2019, representing an increase of over 40% in this metabolic burden over the past three decades (13). This trend indicates the need to synthesise current evidence on how dietary sugar, metabolic imbalance, and inflammation interact to promote bladder cancer development.

Experimental evidence from *in vitro* and animal studies offers insights into how elevated glucose levels can directly influence bladder cancer biology by promoting tumour growth, invasion, and resistance to therapy (14–16). Epidemiological studies have suggested associations between high dietary glycaemic index (GI) and/or glycaemic load (GL) (17–19), and excessive consumption of sugary drinks (20–22) with bladder cancer risk, though evidence remains inconsistent. Importantly, adopting a diabetes risk reduction diet (reduced refined sugar intake and high fibre consumption) has been shown to decrease the risk of bladder cancer by more than 20% (23). This is particularly significant considering that a high-sugar diet is a major contributor to chronic low inflammation, which is a well-known driver of cancer (24). A high glucose environment stimulates the production of pro-inflammatory cytokines, promotes oxidative stress, and alters the gut, and potentially the urinary, microbiota, all of which can enhance carcinogenic signalling in the bladder (25, 26). Ultimately, chronic inflammation, both systemic and localised within the bladder microenvironment, plays a critical role in bladder cancer development and progression. Persistent inflammatory states activate inflammatory mediators and cellular pathways facilitating tumour initiation, angiogenesis, invasion, and metastasis (27).

Overall, these findings emphasise a bidirectional relationship in which high sugar consumption not only promotes bladder cancer through metabolic substrates, but also reshapes the inflammatory landscape, further promoting tumour growth and immune evasion. As bladder cancer continues to challenge clinicians due to high recurrence rates and limited therapeutic response, addressing diet-induced inflammation could be a promising approach to improve treatment and outcomes.

This review aims to synthesise current evidence on the complex relationships among dietary sugar intake, gut microbiota dysregulation, systemic inflammation, and bladder cancer, while also highlighting potential dietary interventions for prevention. It consolidates experimental findings, focusing on mechanistic insights from *in vitro* and *in vivo* studies, and summarises relevant epidemiological evidence.

## 2 Sugar metabolism and cancer

Changes in sugar metabolism are closely associated with disease development, particularly cancer. The reprogramming of glucose metabolism, known as the Warburg effect, is a well-known hallmark of cancer. It describes how cancer cells preferentially convert glucose to lactate via glycolysis, even in the presence of sufficient oxygen (28, 29). Even though this metabolic reprogramming is much less efficient in ATP production than oxidative phosphorylation, it generates glycolytic intermediates, including glucose-6-phosphate, 3-phosphoglycerate, phosphoenolpyruvate, and pyruvate that are necessary for nucleotide, amino acid, and lipid synthesis in cancer cells (30). In addition, it also promotes cancer invasion because of the acidification of the surrounding microenvironment which results from the accumulation of large amounts of lactic acid and protons (30, 31). Evidence has shown that a low extracellular pH facilitates the breakdown of the extracellular matrix within the tumour microenvironment (TME), induces apoptosis of normal cells, and promotes the survival and adaptation of cancer cells under acidic conditions (32, 33). Moreover, acidification also reduces the activity of CD8+ tumour-specific effector T cells (34, 35) and natural killer (NK) cells (36), thereby contributing to immune evasion and tumour progression. It also promotes the recruitment of M2-macrophages (37, 38) and regulatory T cells (39), which further contribute to the immunosuppressive microenvironment.

In addition to the Warburg effect, dietary sugars can influence how cancer cells produce and use energy. Emerging epidemiological studies suggest that high dietary sugar intake is associated with an increased risk of several cancers (40–42). Excessive consumption of refined sugars leads to elevated levels of glucose and insulin in the bloodstream (43). Hyperglycaemia and hyperinsulinaemia can indirectly increase tumour growth by providing substrates for glycolysis while also activating insulin and insulin-like growth factor (IGF) signalling pathways that promote proliferation of tumour cells (44). Furthermore, excess glucose also generates more metabolic products, such as lactate, that acidify the TME, promoting cancer progression, immune evasion, and therapy resistance (45, 46).

Besides glucose, fructose has also been implicated in supporting tumour growth (47, 48). Fructose enters cells primarily via the high-affinity fructose transporter GLUT5, which is frequently upregulated in several cancers, including breast (49), colorectal (50), and ovarian cancer (51). The expression of GLUT5 is regulated by factors such as hypoxia-inducible factor 1-alpha (52) and inflammatory signalling via the IL-6/STAT3 axis (53), highlighting its role in tumour adaptation and progression. Once internalized, fructose is rapidly phosphorylated by ketohexokinase (KHK) to fructose-1-phosphate, bypassing the rate-limiting step of glycolysis. KHK exists in isoforms, including KHK-A, which has been shown to have additional pro-tumoral kinase activities facilitating cancer growth and metastasis. Aldolase B (ALDOB) further metabolizes fructose-1-phosphate, feeding into glycolysis, the TCA cycle, and providing substrates for *de novo* lipogenesis (54). Recent evidence suggests that in combination with glucose, fructose elevates the NAD^+^/NADH ratio, which in turn promotes the invasiveness of colorectal cancer cells (55).

Taken together, the interplay between cancer cell–intrinsic metabolic rewiring and the external availability of dietary sugars fundamentally shapes tumour biology. A deeper understanding of these interactions is important for interpreting experimental and clinical evidence and has potential implications for therapeutic strategies and dietary interventions, as discussed in the following chapters.

## 3 Glucose metabolism dysregulation in bladder cancer cells: mechanisms and therapeutic targeting potential from preclinical studies

Preclinical studies are essential for elucidating mechanistic pathways that cannot be examined directly in human tissues. This chapter synthesizes evidence on glucose-driven signalling in bladder cancer and evaluates therapeutic approaches targeting these pathways. While epidemiological studies demonstrate associations between dietary patterns and bladder cancer incidence (discussed in the next chapter), only *in vitro* and *in vivo* investigations can reveal the biological mechanisms by which glucose dysregulation activates specific signalling cascades in urothelial cells.

A consistent body of experimental evidence demonstrates that elevated glucose levels can directly fuel aggressive behaviours in bladder cancer cells (**Table 1**). The proliferation of bladder cancer cell lines has been shown to increase with exposure to higher glucose concentrations compared to physiological levels (56). Contrarily, low doses of glucose resulted in reduced proliferation (56). This correlation has been linked to up-regulation of pyruvate kinase M2 (PKM2), a key glycolytic enzyme. The clinical relevance of PKM2 was established through urine biomarker studies. Remarkably, 90% of bladder cancer urine samples showed elevated tumour M2-PK levels compared to normal controls, identifying PKM2 as both a mechanistic driver and potential diagnostic marker. Additionally, targeted inhibition of PKM2 using shikonin, a natural PKM2 inhibitor, reduced cell proliferation and switched PKM2 isoforms from the inactive dimer to the active tetramer form, demonstrating the therapeutic potential of metabolic targeting (56). Furthermore, studies have shown that exposing bladder cancer cells to high glucose concentrations results in a dose-dependent increase in cell proliferation compared with normal glucose concentrations (14). This study identified the Wnt/β-catenin signalling pathway as a key mediator of glucose-induced proliferation. High glucose significantly upregulated Wnt-5a and β-catenin mRNA and protein expression, with a linear correlation between Wnt/β-catenin pathway activation and bladder cancer cell proliferation. The study also demonstrated that high glucose significantly promoted colony formation of bladder cancer cells compared to the control groups, providing evidence for the effects of glucose on tumorigenic potential beyond simple proliferation (14).

**Table 1:** Effects of high glucose conditions in bladder cancer preclinical models.

In vitro / In vivo model	Glucose levels (mM / mg/dL)	Observed effects in high glucose conditions	Mechanism / pathways activated by high glucose	Ref.
*In vitro:* HTB-9, HTB-5, UM-UC-3	Low: 1.39 mM / 25 mg/dL; Normal: 5.6 mM / 100 mg/dL; High: 11.1 mM / 200 mg/dL	Increased/reduced proliferation of cancer cells in high/low doses.	PKM2 upregulation. Shikonin (PKM2 inhibitor) reduces cell proliferation.	(56)
*In vitro:* T24	Normal: 5.5 mM / 99 mg/d; High: 10, 20, 30 mM / 180, 360, 540 mg/dL	Dose-dependent increase in proliferation and cell colony formation.	Wnt/β-catenin pathway activation.	(14)
*In vitro:* T24, UM-UC-3 *In vivo:* STZ-induced T24 xenograft nude diabetic mice	*In vitro:* Low: 2.8 mM / 46.5 mg/dL; Normal: 11.2 mM / 201.8 mg/dL; High: 25 mM / 450 mg/dL; *In vivo:* Euglycaemic: <16.7 mM / 300.7 mg/dL; Hyperglycaemic: ≥30 mM / 540 mg/dL	*In vitro:* Increased proliferation, invasion, EMT and autophagy in high glucose conditions and vice versa under low glucose conditions. *In vivo:* Increased tumor growth and metastasis, decreased survival of animals, increased EMT, decreased autophagy.	Increased expression of YAP1/TAZ via AMPK. YAP1 and TAZ regulate GLUT1 expression. Treatment with metformin inhibited EMT, YAP1, TAZ and GLUT1 expression.	(15, 57)
*In vitro:* T24, HT1376	Normal: 5 mM / 90.1 mg/dL; High: 30 mM / 540 mg/dL	Increased proliferation and invasion of cancer cells, upregulation of EMT markers.	Downregulation of tumor suppressor GDF15 via Akt and AMPK activity. Metformin and CAPE can rescue GDF15 and reduce the proliferation and invasion of cancer cells.	(16)
*In vitro:* BFTC-905	Normal: 5 mM / 90.1 mg/dL; High: 25 mM / 450.4 mg/dL	Increased migration and invasion of cancer cells.	Migration and invasion could be inhibited by laminar shear stress.	(58)
*In vitro:* 5637, T24	Normal: not specified; High: 20 mM / 360 mg/dL and 40 mM / 720 mg/dL	Downregulation of YTHDC1, upregulation of GLUT3 and RNF183, increased glucose uptake.	YTHDC1/GLUT3/RNF183 positive feedback loop.	(59)

PKM2, pyruvate kinase M2; STZ, streptozotocin; EMT, epithelial-mesenchymal transition.

High levels of glucose have been shown to promote bladder cancer progression *in vitro* by enhancing epithelial‐mesenchymal transition (EMT), increasing invasiveness, and inhibiting autophagy compared to normal glucose levels, with the opposite effects at low glucose levels (15, 57). Mechanistically, studies showed that high glucose regulates the YAP1/TAZ (Yes-associated protein 1/Transcriptional coactivator with PDZ-binding motif) pathway through AMP-activated protein kinase (AMPK) signalling. YAP1 and TAZ serve as central effectors of the conserved Hippo pathway that governs carcinogenesis, regeneration, and metabolism. The studies found that YAP1 and TAZ modulate autophagy markers, facilitate EMT, and regulate GLUT1 expression, a key glucose transporter in bladder cancer cells (15, 57). These *in vitro* findings were confirmed *in vivo* using a streptozotocin (STZ)-induced diabetic mouse model (15, 57). STZ, a glucosamine-nitrosourea compound that selectively destroys pancreatic β-cells via GLUT2-mediated uptake, produces sustained hyperglycaemia. When maintained on a high-glucose diet, hyperglycaemic mice bearing bladder cancer cell xenografts exhibited significantly greater tumour volume, accelerated metastasis, and shorter survival than euglycaemic controls, which received insulin to maintain euglycemia (15, 57). Tumours from hyperglycaemic mice showed higher expression of YAP1 and TAZ, increased levels of EMT markers, and reduced autophagy, directly demonstrating the clinical relevance of glucose dysregulation in bladder cancer progression (15, 57). Moreover, analysis of human bladder cancer specimens revealed that expression of YAP1 and TAZ positively correlates with tumour grade and is further elevated in patients with type 2 DM (T2DM) (15, 57), highlighting the pivotal role of the YAP1/TAZ pathway in glucose-driven oncogenesis.

Another study performed by Chang et al. (16) confirmed that treatment with high glucose enhanced proliferation and invasion of bladder cancer cells and upregulated EMT markers such as N-cadherin, snail, slug, and vimentin. The study also showed that under high glucose conditions, growth differentiation factor 15 (GDF15), a cytokine and member of the TGFβ superfamily with tumour-suppressor functions in bladder cancer, was downregulated. Conversely, induction of GDF15, for example by the antidiabetic drug metformin or the natural compound caffeic acid phenethyl ester (CAPE), attenuates bladder cancer cell proliferation and invasion (16). High glucose inhibits the beneficial effects of metformin or CAPE on AMPK activity, thereby suppressing GDF15 expression. This suggests that high glucose environments may undermine endogenous tumour suppressive mechanisms in bladder cancer (16). Therefore, AMPK activation emerges as a key therapeutic strategy to counteract glucose-driven inflammation and cancer progression.

A study by Lee et al. (58) revealed that high glucose levels induce migration and invasion of bladder cancer cells. This process can be effectively inhibited by applying laminar shear stress, a flow-induced mechanical force generated by interstitial, blood, and/or lymphatic fluid flows in cancer cells during metastasis (mimicked *in vitro* by continuously perfusing the cells with appropriate medium) (58). The results of the study suggest that laminar shear stress may serve as a feasible tool to reduce bladder cancer motility in diabetics (58).

Yan et al. (59) identified a novel YTHDC1/GLUT3/RNF183 positive feedback loop regulating glucose metabolism and bladder cancer progression in bladder cancer cell lines under hyperglycaemic conditions. The study demonstrated that hyperglycaemia downregulates YTHDC1, an m6A reader protein that normally suppresses GLUT3 expression. The loss of YTHDC1 function creates a self-perpetuating cycle: reduced YTHDC1 leads to GLUT3 upregulation, which in turn enhances glucose uptake and promotes bladder cancer progression. Notably, GLUT3 was found to destabilize YTHDC1 by upregulating the RNF183 expression, a hypertonicity-responsive ubiquitin ligase, thereby completing the feedback loop. This mechanism provides a molecular explanation for how chronic hyperglycaemia enhances glycolytic capacity in bladder cancer cells and establishes a connection between glucose stress and m6A RNA methylation modifications. Additionally, YTHDC1 was demonstrated to be downregulated in bladder cancer tissue and associated with poor cancer prognosis (59).

In addition to mechanistic analysis, preclinical studies have demonstrated that targeting glucose metabolism can be exploited as a therapeutic option for bladder cancer (**Table 2**). The therapeutic targeting of glucose metabolism has been validated in preclinical *in vitro* models using 2-deoxy-D-glucose (2DG), a synthetic non-metabolizable glucose analog that acts as a hexokinase 2 (HK2) inhibitor, targeting one of the rate-limiting enzymes in glycolysis (60). Treatment with 2DG significantly reduced the viability, proliferation, migration, and invasion of several bladder cancer cell lines, while promoting apoptosis. 2DG reduced extracellular lactate production, confirming inhibition of glycolytic activity (60). *In vitro* findings were validated *in vivo* using the chick chorioallantoic membrane assay, which demonstrated significant reductions in both tumour growth and angiogenesis after 2DG treatment (60). In bladder cancer patients, the study identified HK2 as an independent prognostic factor for disease-free and overall survival. High HK2 expression was associated with more aggressive tumour features and poorer patient outcomes, supporting its role as a prognostic biomarker in bladder cancer (60).

**Table 2:** Therapeutic targeting of glucose metabolism in preclinical bladder cancer models. Abbreviations: 2DG: 2-deoxy-D-glucose, HK2: hexokinase 2.

In vitro/ In vivo model	Treatment	Observed effects	Mechanism/pathways activated by treatment	Ref.
*In vitro:* T24, HT1376, KU1919 *In vivo:* Chick chorioallantoic membrane	Treatment of cancer cells with 2DG, a HK2 inhibitor.	*In vitro:* Reduced viability, proliferation, migration and invasion of cancer cells increased apoptosis and cell cycle arrest, decreased extracellular lactate production and glycolysis. *In vivo:* Reduction of tumour growth and angiogenesis.	Inhibition of glycolysis through HK2 targeting, disruption of glucose metabolism, and potentiation of cisplatin efficacy in cisplatin-resistant cells.	(60)
*In vitro:* 5637, UM-UC-2	Treatment of cancer cells with metformin.	Reduced proliferation (dose/time-dependent), suppressed lactate production and glycolysis.	Downregulation of ncRNA UCA1, inhibition of mTOR-STAT3-HK2 pathway.	(61)

A study by Li et al. (61) revealed the mechanism underlying the therapeutic effects of the antidiabetic drug metformin in bladder cancer cells *in vitro*. Metformin inhibited bladder cancer cell proliferation in a dose- and time-dependent manner (61). The study demonstrated that metformin downregulates the expression of the long non-coding RNA UCA1 in both a dose- and time-dependent manner, which subsequently inhibits the mammalian target of rapamycin–signal transducer and activator of transcription 3–hexokinase 2 (mTOR-STAT3-HK2) signalling pathway. These findings provide evidence that metformin inhibits cancer cell glycolysis through lncRNA-mediated regulation (61). The potent anti-tumour effects of metformin in bladder cancer were also demonstrated *in vivo*, with reduced growth of tumour xenografts in nude mice (62), supporting its preclinical potential as a glucose-metabolism-targeting therapeutic.

Collectively, these preclinical studies establish that elevated glucose drives inflammation-related pathways in bladder cancer through combined dysregulation of PKM2, YAP1/TAZ, YTHDC1/GLUT3, and HK2, among other nodes. Targeted modulation of these pathways, through both: (i) pharmacological interventions (2DG, metformin) and (ii) mechanistic manipulation (AMPK activation, GDF15 restoration), has been shown to suppress bladder cancer cell behaviour. These findings support the concept that glucose dysregulation is a targetable weakness in sugar-driven inflammation and bladder carcinogenesis.

## 4 Epidemiological studies linking dietary sugars and hyperglycaemia to bladder cancer

Epidemiological data summarized in **Table 3** suggest that chronic hyperglycaemia, whether from elevated dietary GI or DM, is associated with increased bladder cancer risk, while evidence for GL and sweet beverage consumption remains inconclusive.

**Table 3:** **Summary of (meta)analyses of epidemiologic studies on carbohydrate metabolism, glucose regulation, and bladder cancer risk.** This table synthesizes the epidemiologic literature examining associations between carbohydrate quality and quantity, simple sugars, glucose metabolism, and bladder cancer risk. Studies are organized by exposure category. Effect sizes are reported as relative risks (RR), Odds ratio (OR), hazard ratios (HR) or standardized incidence ratio (SIR) with 95% confidence intervals (CI). Abbreviations: BC, bladder cancer; GI, glycaemic index; GL, glycaemic load; DM, diabetes mellitus; FPG, fasting plasma glucose, BMI, body mass index.

Exposures studied	Study design	Population/ sample size/no. of included studies	Effect size for BC risk	Sex-specific findings	Key adjustments	Notes	Ref.
CARBOHYDRATE QUALITY and QUANTITY							
GI, GL	Meta-analysis of cohort and case-control studies	4 studies (2 case-control and 2 prospective cohort).	**GI:** positive association (RR = 1.25, 95% CI: 1.11–1.41, n **=** 4 studies). **GL:** null association (RR = 1.10, 95% CI: 0.85–1.42, n **=** 4 studies).	Not specified	Study-specific adjustments; major confounders: age, sex, smoking, alcohol, BMI, energy intake, education, study centre.	Consistent findings across studies.	(18)
GI, GL	Meta-analysis of prospective cohort studies	3 prospective cohort studies.	**GI:** positive association (RR = 1.23, 95% CI: 1.09–1.40, n **=** 3 studies). **GL:** null association (RR = 0.98, 95% CI: 0.80–1.21, n **=** 3 studies).	Not specified	Study-specific adjustments; age, sex, smoking, BMI, energy, physical activity.	Consistent findings across studies. Low certainty of evidence.	(70)
GI, GL, Total carbohydrates consumption	Meta-analysis of case-control and cohort studies	12 studies (7 case-control and 5 prospective cohort).	**GI:** positive association (pooled OR = 1.25, 95% CI: 1.11–1.41, n **=** 4 studies). **GL:** null association (OR = 1.10, 95% CI: 0.85–1.42, n **=** 4 studies). **Total carbohydrates:** null association (OR = 1.04, 95% CI: 0.92–1.17, n **=** 9 studies).	Not specified	Study-specific adjustments: age, sex, smoking, BMI, energy intake.	Consistent findings across studies. Observational design limits certainty.	(19)
Simple sugars (sweetened beverages)							
Sugary drink consumption	Prospective cohort	Japanese adults; n = 73,024 (112 BC cases).	**Primary analysis** (overall): null association (HR = 1.01 per 100 ml/day; 95% CI: 0.97–1.06).	**Sensitivity** **analysis**: effect only in women; only in continuous analysis, after excluding early cases (>3 years follow-up); HR = 1.11 (95% CI: 1.01 – 1.22) per 100 mL/day increase.	Age, BMI, smoking, physical activity, energy, diabetes, hypertension, alcohol, foods, height.	Primary analysis null with consistent results. Sensitivity analysis (excluding early cases) yields inconsistent results.	(20)
Sweet beverages pattern (high intakes of coffee, tea, and added sugar)	Case-control (factor analysis)	Uruguayan population; n = 756 (255 BC cases, 501 controls; hospital-based).	Strong association (OR = 3.27, 95% CI: 1.96–5.45).	Not specified	Age, sex, residence, urban-rural, education, family history, occupation, BMI, smoking, mate, energy.	Strong dose-response consistency (p<0.0001). Hospital-based case-control study with acknowledged selection and recall bias.	(72)
Soft drinks consumption (continuous and categorical)	Prospective cohort (EPIC)	n = 233,236 participants (513 BC cases).	**Continuous intake** (per 100 mL/day): weak positive association for all BC cases (HR = 1.04, 95% CI: 1.00–1.08) and low-risk BC cases (HR = 1.07, 95% CI: 1.02–1.11). **Categorical analysis**: association not confirmed (all BC cases: HR = 1.09, 95% CI: 0.88–1.35; low-risk BC cases: HR = 1.33, 95% CI: 0.97–1.83). **Stratified analysis by smoking status**: no differences were found.	Not specified	Age, sex, centre, energy intake, smoking status, duration, and intensity of smoking.	Single large study. Weak effect may be by chance. Categorical analysis contradicts continuous analysis.	(21)
Glucose metabolism							
Risk/incidence studies							
DM	Meta-analysis of cohort studies	21 cohort studies (16 incidence; 7 mortality studies)	**Overall:** positive association (RR = 1.23, 95% CI: 1.12–1.35, n = 15 studies). **Incidence:** RR = 1.21 (95% CI: 1.09–1.35). **Mortality:** RR = 1.25 (95% CI: 1.17–1.35).	**Men**: RR = 1.23, 95% CI: 1.06–1.42, n = 10 studies; significant. **Women**: RR = 1.24, 95% CI: 0.95–1.61; n = 6 studies; not significant.	Study-specific adjustments (typical across studies: age, sex, smoking status, BMI, physical activity, alcohol, education, ethnicity, follow-up duration).	Smoking adjustment was key to understanding association. High heterogeneity overall. Mixed publication bias results.	(78)
DM (type 2 primarily; mixed type 1 + 2 in some studies)	Meta-analysis of cohort studies	29 cohort studies (18 incidence studies, 11 mortality studies).	**Incidence:** positive association (RR = 1.29, 95% CI: 1.08–1.54, n = 18 studies). **Mortality:** positive association (RR = 1.33, 95% CI: 1.14–1.55, n = 11 studies). **Smoking adjusted studies:** Incidence: association remains (RR = 1.33, 95% CI: 1.19–1.47, n = 7 studies). Mortality: association remains (RR = 1.29, 95% CI: 1.19–1.39, n = 5 studies). **BMI adjusted studies:** Incidence: similar with/without adjustment. Mortality: Stronger with adjustment (RR 1.35 vs 1.31).	**Incidence:** **Men:** RR = 1.36; 95% CI: 1.05–1.77; n = 10 studies; significant. **Women:** RR = 1.28, 95% CI: 0.75–2.19, n = 6 studies; not significant. **Mortality:** **Men:** RR = 1.54, 95% CI: 1.30–1.82, n = 5 studies; significant. **Women:** RR = 1.50, 95% CI: 1.05–2.14, n = 3 studies; significant.	Study-specific adjustments (typical across studies: age, sex, smoking status, pack-years, BMI, alcohol, education, ethnicity, follow-up duration).	High heterogeneity, partially explained by smoking adjustment and geographic region. Sensitivity analyses demonstrated robustness of positive associations in both incidence and mortality endpoints. No significant publication bias detected.	(77)
DM (mixed type 1 and 2)	Meta-analysis of observational studies	24 studies (10 case-control and 14 cohort).	**Overall:** positive association (RR = 1.30, 95% CI: 1.18–1.43, n = 24 studies). **Cohort studies:** positive association (RR = 1.23, 95% CI: 1.09–1.37, n = 14 studies). **Case-control studies:** positive association (OR = 1.46, 95% CI: 1.20–1.78, n = 10 studies). **Smoking adjusted studies:** positive association (RR = 1.34, 95% CI: 1.19–1.51, n = 16 studies). **BMI adjusted studies:** positive association (RR = 1.17, 95% CI: 1.03–1.34, n=10 studies). **Glucose-lowering drugs:** borderline/null association (RR = 1.57, 95% CI: 0.96–2.55, n = 3 studies). **Insulin therapy**: borderline/null association (RR = 1.52, 95% CI: 1.00–2.32, n = 3 studies).	**Overall:** **Women:** RR = 1.23, 95% CI: 1.02–1.49, n = 5 studies; significant. **Men:** RR = 1.07; 95% CI: 0.97–1.18, n = 10 studies; not significant.	Study-specific adjustments (typical across studies: age, sex, smoking status, BMI, alcohol, occupational exposure).	Type 1 DM studies included but excluding them did not change results. Follow-up duration critical: in cohort studies <10 years follow-up did not show an association (RR = 1.30, 95% CI: 0.91–1.47), while ≥10 years follow-up did show association (RR = 1.17; 95% CI 1.04–1.31).	(79)
DM	Meta-analysis of observational studies	17 studies (13 cohort, and 4 case-control).	**Overall HR:** positive association (RR = 1.15, 95% CI: 1.07–1.24, n = 9 studies). **Pooled SIR:** positive association (RR = 1.19, 95% CI: 1.06–1.34, n = 1 study). **Pooled OR:** positive association (RR = 2.17, 95% CI: 1.63–2.89, n = 3 studies). **Pooled RR:** null association (RR = 1.10, 95% CI: 0.90–1.34, n = 4 studies). **Cohort studies:** positive association (HR = 1.16, 95% CI: 1.09–1.23, n = 13 studies). **Case-control studies:** null association (HR = 1.69, 95% CI: 0.96–2.97, n = 4 studies). **DM duration <5 years:** null association (HR = 1.02, 95% CI: 0.86–1.22, n = 6 studies). **DM duration ≥5 years:** positive association (HR = 1.14, 95% CI: 1.01–1.27, n = 5 studies).	**Men:** HR = 1.15, 95% CI: 1.06–1.24, n = 6 studies; significant. **Women:** HR = 1.08, 95% CI: 0.94–1.24, n = 7 studies; not significant.	Study-specific adjustments: age, BMI, smoking.	Sample size was not reported in several studies, the type of diabetes and mean age were not specified in most studies. No significant publication bias detected.	(80)
High FPG assessed as continuous and categorical variable (prediabetes and diabetes thresholds)	Meta-analysis	34 studies (21 prospective cohorts, 8 case-control design, 3 retrospective cohorts and 2 case-cohorts).	**Overall (FPG level of 7.96 mM vs reference 4.2 mM)**: weak association (RR = 1.25; 95% CI: 1.02–1.52; n = 34 studies). **FPG levels of 6.1 mmol/L (prediabetes)**: borderline positive association (RR = 1.29; 95% CI: 0.97–1.70; n not reported). **FPG levels of 7.0 mmol/L** (**DM diagnosis**): borderline/null positive association (RR = 1.44; 95% CI: 0.96–2.15; n not reported). **Burden of Proof Risk Factor** (exposure-averaged) = 1.02 per 1 mmol/L increase. **Cohort studies only**: null association (RR 1.31; 95% 0.87–1.97; n = 24 studies).	Not specified	Adjusted for different DM definitions, FPG imputation, and ICD-10 outcome verification.	79% of included studies used diabetes status (administrative/self-report) rather than direct FPG measurement, which may explain weaker associations in FPG meta-analysis. No evidence of publication bias. Weak certainty.	(85)
Prognostic studies (outcomes in patients with existing bladder cancer)							
DM (prognostic factor in established BC)	Meta-analysis of cohort studies	9 studies (8 retrospective cohorts and 1 prospective cohort).	**Cancer-specific mortality:** positive association (HR = 1.67, 95% CI: 1.29–2.16, n = 7 studies). **Disease progression:** positive association (HR = 1.54, 95% CI: 1.15–2.06, n = 8 studies). **Disease recurrence:** positive association (HR = 1.40, 95% CI: 1.32–1.48, n = 8 studies).	Not specified	Study-specific adjustments (common across studies: age, tumor characteristics (grade, stage, type)).	The retrospective design of 8 studies was noted as a potential source of bias. Examines outcomes after BC diagnosis. No evidence of publication bias.	(81)
DM (prognostic factor post-radical cystectomy)	Meta-analysis of cohort studies	5 studies (4 retrospective and 1 prospective).	**Overall survival:** positive association (HR = 1.36, 95% CI: 1.30–1.43, n = 5 studies). **Cancer-specific survival:** positive association (HR = 1.59, 95% CI: 1.29–1.95, n = 2 studies). **Disease recurrence**: positive association (OR = 1.76, 95% CI: 1.25–2.49, n = 2 studies).	Not specified	Study-specific adjustments (age, gender, follow-up time, comorbidities, and tumor stage).	No publication bias detected. High sensitivity and specificity confirmed.	(82)

Bold text highlights the primary findings or key outcomes reported in each study.

High GI and GL foods have been shown to raise blood glucose levels even in the absence of diabetes, obesity and metabolic syndrome. GI measures a food’s carbohydrate content by its effect on postprandial (after-meal) blood glucose concentrations, while GL combines both the quality (GI) and quantity of carbohydrates (mean GI multiplied by total carbohydrate) within a single serving (63). Consuming meals with high GI or GL values leads to a greater increase in blood glucose levels than low GI or GL meals (64, 65). Conversely, a diet low in GI or GL has been associated with reduced inflammation and a reduced risk of T2DM (66, 67). Despite inconsistencies among studies, several investigations have established a positive correlation between high GI and/or high GL diets and bladder cancer risk (17–19). It has been proposed that chronic consumption of a high GI/GL diet leads to consistently high blood glucose levels, and consequently chronically elevated insulin concentrations (18). Repeated high postprandial glucose and insulin spikes from high GI/GL diets activate insulin-like growth-factor-I (IGF-1) signalling pathways involved in malignant transformation, tumour progression through multiple intracellular pathways (AKT, MAPK) and resistance to apoptosis (68, 69).

An Italian case-control study (17) showed a direct association between bladder cancer risk and high dietary GL, as well with consumption of refined carbohydrate foods such as bread and pasta. These associations appeared to be stronger in individuals with low vegetable consumption. A subsequent meta-analysis of cohort and case-control studies that incorporated Italian case-control study showed a small increase in the risk of bladder cancer with high GI, based on four included studies (18). A study by Long et al. (70) performed a meta-analysis of three prospective cohort studies and found a positive association between GI and bladder cancer risk, although the certainty of evidence was low. In contrast, no association was observed between GL and bladder cancer risk. One of the studies included in this meta-analysis was the Prostate, Lung, Colorectal, and Ovarian Cancer (PLCO) Screening Trial, which evaluated a total of 101,721 eligible participants aged 55–74 years, and did not find evidence supporting an association between higher GI or GL diets and bladder cancer risk (71). This divergence among the constituent studies indicates some heterogeneity in the evidence base, although the overall meta-analysis summary estimate supported a positive GI-bladder cancer association. Another meta-analysis of 12 observational studies found GI to show a positive linear association with bladder cancer risk (19). Taken together, GI emerges as a more robust and consistently significant predictor of bladder cancer risk.

Limited and inconsistent associations have been found between sugary drink consumption and bladder cancer risk (20–22). Leung et al. (20) found no association between sugary drink consumption and bladder cancer risk in Japanese adults in the primary analysis. However, after excluding cases diagnosed within the first three years (to address reverse causation), they found that sugary drink consumption was positively associated with bladder cancer risk among women. The Uruguayan case-control factor analysis by De Stefani et al. (72) identified the “sweet beverages” pattern, characterized by high intakes of coffee, tea, and added sugar, which showed the strongest association with bladder cancer risk. Ros et al. (21) found a positive association between soft drinks consumption and bladder cancer in the European Prospective Investigation into Cancer and Nutrition (EPIC) study. However, because this observation was limited to a single study and was not observed consistently across analytical methods within that study, it may be attributable to chance. In a broader meta-analysis of 27 observational studies examining sweetened beverage intake and overall cancer risk, Llaha et al. (22) did not find a significant association between sweetened beverages (sugar-sweetened, artificially sweetened, or fruit juices) and bladder cancer risk. This meta-analysis did not identify bladder cancer as a primary outcome for separate analysis (bladder cancer was included in only 6 studies); consequently, it did not provide pooled estimates specific to bladder cancer. The limited epidemiological evidence for sweetened beverages and bladder cancer risk therefore remains inconclusive, with findings restricted to a few individual studies showing divergent results across populations and beverage types.

Importantly, a large prospective analysis within the PLCO Screening Trial was published in 2023 (23) designed to assess whether adherence to a dietary pattern aimed at reducing T2DM risk also influenced bladder cancer incidence. The results showed that adherence to a diabetes risk reduction diet (emphasizing low‐GI foods, high fibre, healthy fats, and minimal sugary beverages and processed meats) was associated with a reduced bladder cancer risk of over 20%. This provides evidence that dietary patterns promoting blood glucose control may be protective, supporting the role of lifestyle‐mediated glycaemic control in bladder cancer prevention (23). Several studies have reported an inverse association between a healthy diet and bladder cancer risk (73–76), which are discussed in more detail in chapter 6.

Elevated blood glucose and DM are characterised by hyperglycaemia and long-term hyperinsulinaemia and have been identified as risk factors for bladder cancer. Although findings from epidemiological studies are controversial, most meta-analyses support DM as a risk factor for bladder cancer, as both the incidence and death rates of bladder cancer increase in diabetics (77–80). Due to the lack of differentiation between type 1 and type 2 diabetes in some studies, the term diabetes mellitus (DM) is used. A recently published meta-analysis in 2025, incorporating 17 observational studies (80), found that bladder carcinoma risk was worsened by DM, with male gender and DM duration of ≥5 years being among the significant risk factors. Beyond its role as a risk factor for bladder cancer development, DM also serves as a prognostic factor that worsens outcomes in patients with established bladder cancer. A meta-analysis that included 9 studies (retrospective and prospective) revealed that DM patients demonstrate significantly worse outcomes in bladder cancer, with increased risks for cancer-specific mortality, disease progression, and recurrence compared to non-diabetics (81). A meta-analysis of the impact of DM on the prognosis of bladder cancer patients after radical cystectomy, which included five studies, found that DM increases both overall and tumour-specific death (82). Li et al. (15) conducted a retrospective study of bladder cancer patients with normal glucose tolerance and T2DM, with histopathological analysis of bladder cancer tissue samples. The study revealed a higher pathological grade and more advanced stage in patients with bladder cancer with comorbid T2DM (15). Similarly, Li et al. (57) showed that glucose levels and T2DM were positively associated with pathological grade and tumour-node-metastasis (TNM) stage in bladder cancer. These studies provide direct clinical evidence that glucose dysregulation is associated with more aggressive pathological features of bladder cancer. A recent bioinformatics and molecular study by Ma et al. in 2025 (83) has identified CXCL12 as a key gene connecting bladder cancer and DM. The study found that, under elevated blood glucose conditions, CXCL12 is associated with aggressive tumour characteristics, specifically: increased proliferation and invasive capacity, induction of EMT, activation of cancer-associated fibroblasts (CAF), and immune suppression within the tumour microenvironment. Together, these changes promote tumour progression and invasiveness.

High FPG has been identified as an independent risk factor for bladder cancer. A 10-year follow-up prospective Korean study by Jee et al. (84) included more than a million people and found significantly higher mortality for bladder cancer in men with blood glucose levels >6.9 mM, after adjusting for smoking, age and alcohol consumption. A 2025 meta-analysis by Teixeira et al. (85), which included Korean study by Jee et al. (84) performed the most conservative and systematic assessment of the evidence to date, which included 34 studies, and found a weak relationship between high FPG and bladder cancer risk. A study by Wu et al. (13) estimated the burden of bladder cancer attributable to high FPG from 1990 to 2019. FPG was defined as any concentration above the theoretical minimum risk exposure level of 4.8–5.4 mM. This criterion includes any high FPG conditions including diabetes, which is diagnosed at levels of 7 mM or higher. The study revealed a substantial global increase in the burden associated with elevated FPG, with age-standardized death rates rising by 39.18% and disability-adjusted life years rising by 41.48% over the 30-year period. Notably, the impact was more pronounced in regions with high socio-demographic indexes (13). The study highlighted the growing role of metabolic risk factors in bladder cancer mortality and morbidity globally. Monitoring FPG levels among patients with bladder cancer is critical to lower the corresponding burden.

## 5 Sugar intake and chronic inflammation in bladder cancer

Excessive dietary sugar intake triggers systemic inflammation through metabolic alterations in insulin-sensitive tissues and immune system dysregulation, characterized by elevated production of pro-inflammatory mediators and altered immune cell function (86). Clinical and experimental studies have shown that diets rich in sugar-sweetened beverages elevate blood lipids, fasting glucose, and high-sensitivity C-reactive protein (hs-CRP), while promoting the production of pro-inflammatory mediators. Fructose and sucrose appear to exert stronger pro-inflammatory effects than glucose (87). Controlled trials report that even moderate consumption of sugar-sweetened beverages significantly alters LDL particle profiles and inflammatory biomarkers (88). Experimental models confirm these findings, demonstrating that fructose induces visceral adipose tissue inflammation and enhances expression of pro-inflammatory cytokines such as IL-1β, IL-6, and TNF-α, alongside activation of NF-κB and related signalling pathways (89). Hyperglycaemia further enhances immune cell dysregulation. Chronic high blood glucose sensitizes macrophages to produce IL-1β, IL-6, and TNF-α through NF-κB and MAPK activation (25, 90), while impairing neutrophil function (91) and driving T cell polarization toward pro-inflammatory Th1 and Th17 phenotypes (92).

**Figure 1:**
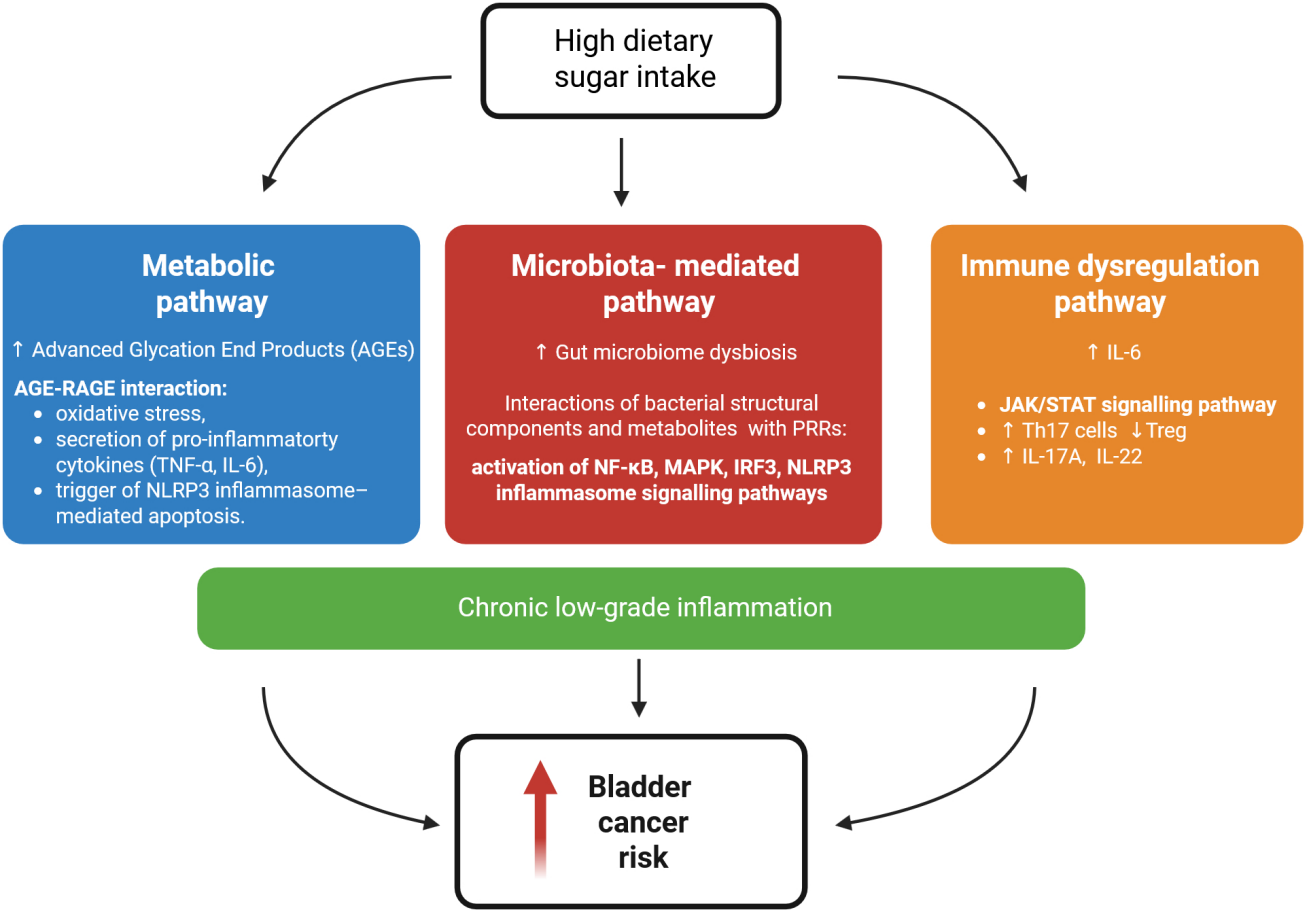
Proposed mechanisms linking high dietary sugar intake to increased bladder cancer risk. High sugar consumption contributes to chronic low-grade inflammation through three interconnected signalling pathways: (i) Metabolic pathway: excess sugar promotes the formation of AGEs, which interact with RAGE and, through downstream signalling, induce oxidative stress, increase secretion of pro-inflammatory cytokines (TNF-α, IL-6), and activate the NLRP3 inflammasome; (ii) Microbiota-mediated pathway: high dietary sugar intake alters gut microbiome composition. Microbial components and metabolites engage PRRs and activate pro-inflammatory signalling via NF-κB, MAPK, IRF3, and NLRP3 inflammasome pathways; and (iii) Immune dysregulation pathway: elevated IL-6 activates the JAK/STAT signalling pathway, disrupts the Th17/Treg balance, and increases production of IL-17A and IL-22, thereby skewing the immune system towards a pro-inflammatory state. Together, these processes drive chronic low-grade inflammation, which may in turn increase bladder cancer risk. Figure was created with BioRender.

A key molecular link between sugar intake and inflammation is the accumulation of advanced glycation end products (AGEs). These compounds arise through the non-enzymatic glycation of reducing sugars, such as glucose, fructose, and galactose, with proteins, lipids, and nucleic acids, and act as damage-associated molecular patterns (DAMPs) (93). Through interaction with the receptor for AGEs (RAGE), these compounds were shown to up-regulate the NADPH oxidase pathway and increase ROS/RNS production, thereby amplifying oxidative stress in neutrophils (94). AGE-activated neutrophils also release MPO and neutrophil elastase, which enhance Th1 (IFN-γ) and Th17 (IL-17) differentiation from naïve CD4^+^ T cells (95). Dietary AGEs can stimulate macrophage TNF-α production, secretion of IL-6, and trigger NLRP3 inflammasome–mediated apoptosis (96, 97). AGE-RAGE engagement suppresses IL-2, IFN-γ and IL-10 mRNA expression, while enhancing monocyte IL-6 production via MAPK–ERK and MyD88-NF-κBp50 signalling pathways (98) (**Figure 1**). Importantly, AGE–RAGE-driven inflammatory and oxidative stress responses further upregulate RAGE expression itself, establishing a self-amplifying positive feedback loop. Beyond AGEs, RAGE also recognizes other endogenous ligands, including members of the S100 protein family and high mobility group box 1 (HMGB1), which collectively extend its role as a central hub in chronic inflammation and cancer (99). Both HMGB1 and S100 proteins are strongly implicated in bladder cancer pathogenesis, where they contribute to tumour-associated inflammation, immune evasion, and enhanced migratory and invasive behaviour of cancer cells (100).

High-sugar diets disrupt gut microbiota composition causing gut microbiota dysbiosis. Gut bacteria modulate immune pathways through interactions with pattern recognition receptors (PRRs). Bacterial structural components and metabolites activate receptors such as Toll-like receptors (TLRs), NOD-like receptors (NLRs), and various G protein-coupled receptors (GPCRs) (101). For example, in gut dysbiosis, the overgrowth of Gram-negative bacteria increases luminal lipopolysaccharide (LPS) levels and disrupts the integrity of the intestinal barrier. Circulating LPS activates TLR4, primarily expressed on macrophages and dendritic cells, with the co-receptors cluster of differentiation 14 (CD14) and myeloid differentiation factor 2 (MD2), initiating both MyD88-dependent and MyD88-independent pathways (102). CD14–TLR4–MD2 engagement and TLR4 dimerization recruit TIRAP–MyD88 adaptors, triggering IRAK4-mediated activation and degradation of IRAK1. IRAK1 then associates with TNF-receptor associated factor-6 (TRAF6), leading to TAK1 activation and subsequent induction of the IKK and MAPK pathways. IKK activation promotes NF-κB phosphorylation and nuclear translocation, stimulating expression of COX2, TNF-α, IL-1β, IL-6, and IL-8 (103). In parallel, a second set of TIR domain adaptors TRIF and TRAM mediate the MyD88-independent arm, inducing interferon regulating factor 3 (IRF3) and type I interferon production (104) (**Figure 1**).

Beyond LPS, DAMPs derived from gut microbiota, also activate TLR4 signalling. One such example is oxidized LDL, generated through LPS-driven inflammation and oxidative stress caused by metabolic endotoxemia leads. Oxidized LDL is recognized by CD36 and induces CD36–TLR4–TLR6 heteromerization. This complex activates both MyD88-dependent and independent pathways without MD2 or CD14 and, via NF-κB and ROS, primes the NLRP3 inflammasome (105, 106). Another example includes saturated fatty acid (SFA), which are elevated due to high sugar intake and can be recognized by the CD14–TLR4–MD2 complex, activating inflammatory signalling. SFAs additionally promote dysbiosis and oxidative stress, driving excess LPS and oxLDL production and further engaging CD36–TLR4–TLR6–mediated inflammation (106).

Moreover, fragments of peptidoglycans, a major structural polymer in bacterial cell walls, such as muramyl dipeptide (MDP), bind to the NOD2 receptors, activating RIPK2, which stimulates the NF-κB and MAPK pathways and promote inflammasome assembly, resulting in the maturation of the pro-inflammatory cytokines IL-1β and IL-18 (107, 108).

PRRs on host cells recognise not only bacterial structural components but also act as key mediators for microbiota-derived metabolites. The most extensively studied metabolites of the gut microbiome are SCFAs, such as acetate, propionate, and butyrate (present at ~60:20:20 ratios in the human gut) (109), which signal through distinct G protein-coupled receptors (GPRs), namely GPR109A, GPR41/43 (FFAR2), GPR41 (FFAR3) and Olfactory receptor 78, (OR78), which are expressed in adipose, immune, hepatic, and muscle cells (110). They promote regulatory T-cell differentiation and suppress NF-κB–mediated inflammation, thereby strengthening epithelial barrier integrity (111, 112). Butyrate, in particular, promotes colonic Treg induction through epigenetic Foxp3 up-regulation (113), drives macrophage polarization toward anti-inflammatory M2 phenotype (114), suppresses NF-κB activation, supports epithelial integrity, and activates intestinal gluconeogenesis (115, 116). Another important gut metabolite group are secondary bile acids, such as deoxycholic and lithocholic acid, which modulate immune pathways via FXR, TGR5, and the vitamin D receptor (117). Bile acids regulate antimicrobial peptide production and control pro-inflammatory signalling (118, 119). However, their effects are dependent on context: while certain secondary bile acids suppress inflammation through FXR/TGR5 activation, dysbiosis-associated shifts in bile acid composition can instead promote carcinogenesis by inducing epithelial DNA damage, oxidative stress, and tumour-promoting inflammation (120). Third major group of metabolites are tryptophan-derived microbial metabolites, such as indole-3-aldehyde and indole-3-propionic acid, signal through the aryl hydrocarbon receptor (AhR). AhR induces IL-22 to support epithelial integrity (121) and directs T-cell differentiation in a way that depends on the surrounding immune environment: in anti-inflammatory conditions, AhR promotes regulatory T cells that suppress inflammation, whereas in the presence of pro-inflammatory signals, it promotes Th17 cells that enhance inflammatory responses (122). Together, these receptor-mediated pathways shape the immune cell function, either supporting signalling pathways that maintain intestinal homeostasis or promoting chronic inflammation that can lead to tumorigenesis (123).

Dietary sugar promotes immune dysregulation also by depleting Th17-inducing microbiota, particularly segmented filamentous bacteria (SFB). In mice, high fat diet markedly reduced RORγt^+^Foxp3^-^ Th17 cells and decreased RORγt expression in remaining Th17 cells, indicating loss of functionality, while Th1 cell proportions increased. Loss of SFB strongly correlates with reduced Th17 cells, and sucrose supplementation alone eliminates SFB in a dose-dependent manner, mirroring the Th17 deficits seen with high fat diets (124).

Chronic systemic inflammation has profound implications for bladder carcinogenesis. Clinical evidence consistently links inflammatory markers such as C-reactive protein (CRP) with poor prognosis and therapy resistance in bladder cancer. Elevated pre-treatment CRP levels (above 10 mg/L) predict reduced overall and cancer-specific survival (125–127), while dynamic CRP kinetics during immunotherapy serve as strong predictors of response to immune checkpoint inhibitors (128). Similarly, elevated Systemic Immune-Inflammation Index, integrating neutrophil, platelet, and lymphocyte counts to reflect both systemic inflammation and immune status, significantly correlated with worse overall survival, cancer-specific survival, progression-free survival, and recurrence-free survival in patients with bladder cancer (129). Beyond systemic markers, genetic variations in cytokine signalling pathways, including IL-6 polymorphisms, influence both recurrence risk and responsiveness to intravesical Bacillus Calmette–Guérin (BCG) immunotherapy in non-muscle-invasive bladder cancer (130, 131). These findings underscore the dual role of systemic inflammation as a biomarker and a potential therapeutic target in bladder cancer management.

At the tissue level, the bladder TME exemplifies an inflammation-driven niche that promotes tumour initiation and progression (132). It is characterized by infiltrating immune cells, including macrophages, myeloid-derived suppressor cells, regulatory T cells, dendritic cells, and neutrophils, secreting cytokines such as TNF-α, IL-6, IL-8, and IL-1β (27). These mediators act collectively to sustain chronic inflammation and tumour progression by activating key oncogenic pathways, including COX-2, JAK/STAT3, and NF-κB (27). COX-2 overexpression in bladder cancer stimulates angiogenesis, immune suppression, and tumour proliferation through prostaglandin synthesis (133–135). The JAK/STAT3 axis, activated by IL-6 family cytokines and growth factors, drives malignant transformation of urothelial cells and enhances stem-like properties (136, 137). STAT3 activation in urothelial stem cells has been linked to carcinoma *in situ* and subsequent muscle-invasive disease (138). NF-κB activation correlates with higher histologic grade of bladder cancer (31, 32). Its activation promotes EMT, IL-8 expression, tumour progression, and chemoresistance, whereas NF-κB downregulation enhances chemosensitivity in bladder cancer (139, 140). Together, these pathways create a self-sustaining inflammatory loop that promotes EMT, metastasis, and immune evasion (**Figure 1**).

Additional drivers of bladder inflammation include urinary microbiota and recurrent urinary tract infections (UTIs). Epidemiological data associate recurrent UTIs with increased bladder cancer risk and worse outcomes. Hyperglycaemia and high dietary sugar elevate urinary glucose and osmolality, conditions that favour colonization by uropathogens such as Escherichia coli. This pathogen can persist via biofilm formation and metabolic adaptation, reinforcing chronic infection (141). Chronic cystitis further amplifies COX-2 expression, impairing anti-tumour immunity and promoting angiogenesis (134). Beyond infection-driven inflammation, bacterial genotoxins and metabolites such as hydrogen sulphide exert direct genotoxic effects on urothelial cells (141).

Moreover, oxidative stress represents a critical mechanistic link between hyperglycaemia, inflammation and bladder cancer progression. High glucose levels drive human monocytes and macrophages toward an M1-inflammatory phenotype. *In vitro*, macrophages cultured in hyperglycaemic conditions show increased expression of CD11c and inducible nitric oxide synthase (iNOS), accompanied by reduced expression of arginase-1 and IL-10 (142). Hyperglycaemia also enhanced production of TNF-α, IL-1β, and IL-1Ra, whereas the M2-associated chemokine CCL18 was suppressed by high glucose (143). At the metabolic level, excess glucose oxidation increases the production of electron donors (NADH, FADH_2_) through glycolysis and the TCA cycle, elevating the ATP/ADP ratio and disrupting mitochondrial membrane potential. The resulting high proton gradient impairs electron transfer at complex III, causing electron accumulation in coenzyme Q and promoting superoxide generation. Superoxide is subsequently converted into additional ROS, including hydrogen peroxide, hydroxyl radicals, and peroxynitrite (144). Furthermore, hyperglycaemia can impair antioxidant defences. Upregulation of thioredoxin-interacting protein (TXNIP), via p38-MAPK signalling, inhibits thioredoxin antioxidative activity and further enhances oxidative stress (145). In parallel, the polyol pathway, which can consume over 30% of intracellular glucose under hyperglycaemic conditions, further amplifies oxidative stress. Excess glucose is reduced to sorbitol by the NADPH-dependent enzyme aldose reductase, depleting cellular NADPH. Sorbitol is then oxidized to fructose by sorbitol dehydrogenase, increasing the NADH/NAD^+^ ratio. The resulting NADPH depletion limits regeneration of reduced glutathione, a key antioxidant maintained by glutathione reductase, thereby impairing cellular ROS-scavenging capacity and promoting oxidative stress (146). ROS inflict extensive macromolecular damage, including oxidative DNA lesions such as 8-hydroxy-2′-deoxyguanosine (8-OHdG), which correlate with poor prognosis in bladder cancer (147–149). Elevated ROS also activate redox-sensitive transcription factors like NF-κB and STAT3, reinforcing inflammatory signalling and promoting tumour cell survival. Furthermore, ROS stimulate cytokine release, establishing a feedback loop in which oxidative stress and inflammation perpetuate each other (150). Mitochondrial dysfunction amplifies ROS production, while cancer cells adapt through metabolic reprogramming, particularly the Warburg effect, to sustain redox balance and proliferative advantage under oxidative conditions (148).

Collectively, these findings highlight a complex interplay between dietary sugar, systemic inflammation, urinary microbiota, and bladder-specific inflammatory signalling. This network of metabolic and immunologic disruptions shapes a tumour-promoting microenvironment that drives carcinogenesis, progression, and therapeutic resistance in bladder cancer.

## 6 Dietary patterns, glycaemic control, and microbiome modulation in bladder cancer risk

A well-balanced diet based on whole foods is one of the most effective ways to prevent or reduce chronic inflammation caused by excessive sugar consumption. Whole foods contain a variety of bioactive compounds, such as fibre, essential micronutrients, and phytochemicals, which together have antioxidant, anti-inflammatory, and anti-cancer effects (151). Whole foods also support better regulation of blood glucose and insulin levels (152–154), while a diet high in ultra-processed foods that are high in sugar, unhealthy fats, and salt and low in nutrients (155, 156), has been linked to insulin resistance, high glycaemic responses, and chronic low-grade inflammation (86, 157).

**Figure 2:**
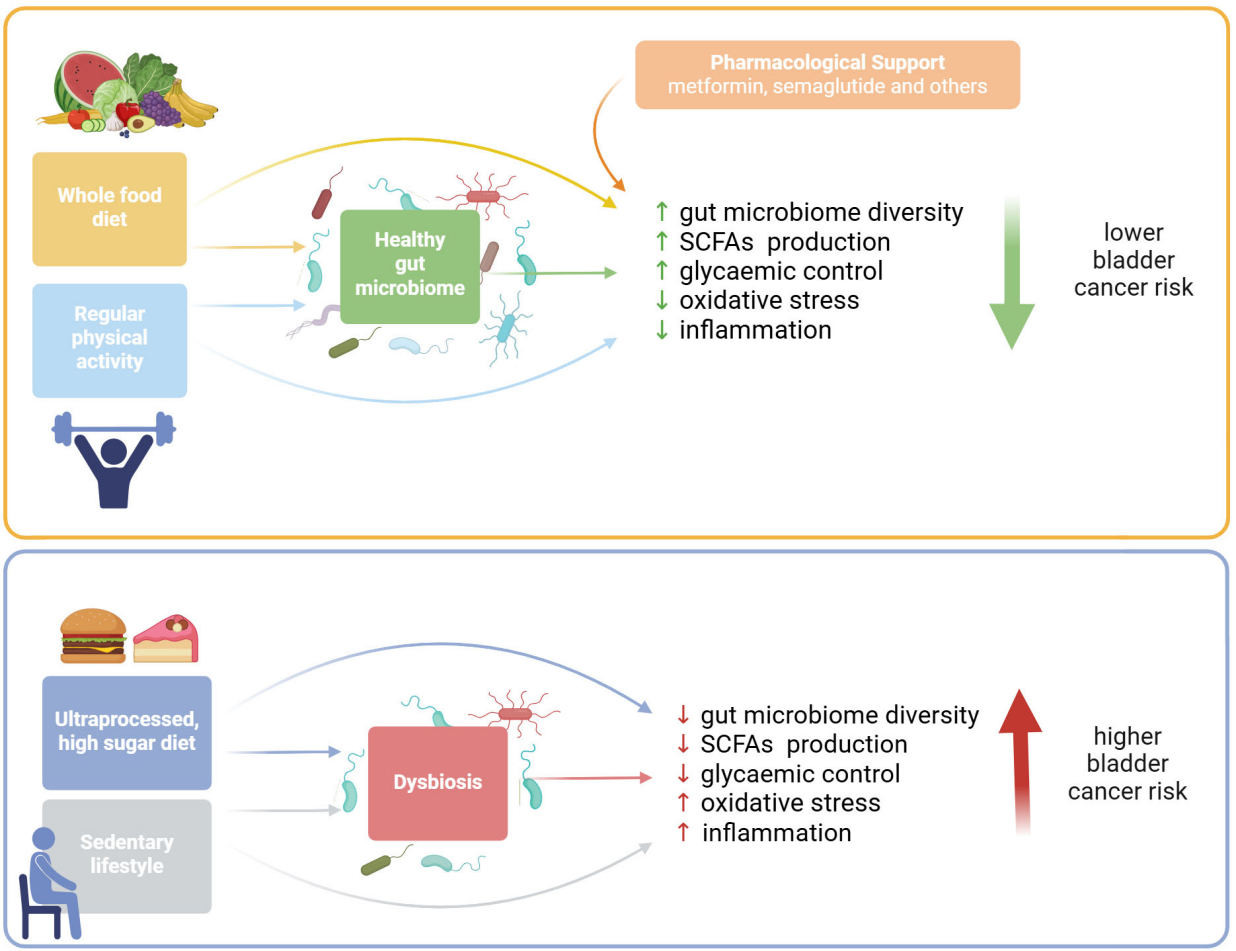
The influence of dietary and lifestyle factors on bladder cancer risk. A healthy whole food diet combined with regular exercise plays a key role in regulating glycaemic control, reducing oxidative stress, and lowering systemic inflammation, both directly and by supporting a balanced gut microbiome. These factors collectively contribute to a reduced risk of bladder cancer. In contrast, a diet high in ultra-processed foods and a sedentary lifestyle can lead to gut microbiome dysbiosis, poor glycaemic control, and chronic low-grade systemic inflammation, all of which are associated with an increased risk of bladder cancer. Figure was created with BioRender.

The Mediterranean diet, a plant-based diet rich in olive oil, fruit, vegetables, legumes, and whole grains, is associated with a lower risk of bladder cancer (73–76, 158) (**Figure 2**). Its protective effect is probably due to the high content of antioxidants such as polyphenols, carotenoids, vitamins C and E, as well as omega-3 and omega-6 fatty acids, which reduce oxidative stress (159) and inflammation (160, 161). Olive oil polyphenols, such as hydroxytyrosol and oleuropein, inhibit the NF-κB pathway by stabilising IκB-α, suppressing IKK activity, and reducing nuclear translocation of NF-κB subunits (162). This suppression decreases transcription of pro-inflammatory cytokines and enzymes, including COX-2, a key enzyme in pro-inflammatory prostaglandin production (163). At the same time, these polyphenols activate the Nrf2–ARE antioxidant pathway, enhancing expression of cytoprotective enzymes and reducing ROS accumulation (164, 165). Omega-3 fatty acids complement these effects by engaging the GPR120/FFAR4–β-arrestin axis to suppress NF-κB activation (166) and by reducing substrate availability for COX-2-mediated prostaglandin synthesis, collectively decreasing inflammatory signalling and oxidative stress (167). Moreover, the Mediterranean diet may help lower GL and GI, contributing to glycaemic control (168–170), while the Western diet, characterised by high consumption of red and processed meat and refined carbohydrates with high GL, is often associated with a higher risk of bladder cancer (171–173). In a review paper, Murillo et al. (174) suggest dietary strategies that reduce the glycaemic response, such as eating foods rich in fibre and protein before carbohydrates, including ingredients such as fibre, fats, proteins, or acetic acid in meals, cooking pasta al dente, and minimising food processing. All of these can contribute to better blood glucose control and lower inflammation.

However, studies on the relationship between diet and bladder cancer can be inconsistent (175, 176), likely due to methodological challenges. Data on diet are usually collected through surveys or questionnaires and may be influenced by the quality of the questionnaire and the memory and honesty of respondents. Despite these limitations, promoting metabolic health through an anti-inflammatory, whole-food diet appears to be a promising strategy for the prevention of inflammation and bladder cancer.

Dietary habits also significantly impact the diversity and activity of the gut microbiota (177). In turn, the gut microbiome plays a crucial role in nutrient extraction, metabolism, and supporting the development and activation of the immune system (178). Diets high in sugar have been shown to reduce bacterial diversity and impair the intestinal epithelial barrier, contributing to low-grade systemic inflammation and, over time, metabolic dysfunction (179). Do et al. (180) demonstrated that mice fed high-glucose or high-fructose diets developed metabolic dysfunction alongside a reduction in gut microbial diversity, marked by a decline in the phylum *Bacteroidetes* and a pronounced expansion of pro-inflammatory *Proteobacteria*. These diets also compromised gut barrier integrity, as shown by decreased expression of the tight junction proteins occludin and ZO-1, increased intestinal permeability, and elevated colonic inflammatory cytokines, including TNF-α and IL-1β. In parallel, the authors observed hepatic inflammation mediated by *Proteobacteria*-derived inflammatory bacterial lipopolysaccharide (LPS), reflected by increased MCP-1, TLR4, and IL-1β expression and lipid accumulation in the liver. Complementarily, Kawano et al. (124) showed that excess dietary sugar disrupts the gut microbiome, causing dysbiosis and loss of key commensal, fibre-fermenting bacteria that normally support intestinal T cell homeostasis. This dysbiosis reduces the population of Th17 cells, leading to decreased secretion of IL-17 and IL-22, increased intestinal lipid uptake, and the development of metabolic syndrome in mice. While animal studies demonstrate rapid and pronounced effects, human data are more variable, particularly in short-term interventions, yet they support similar conclusions. In humans, short-term fructose interventions may induce only minimal changes in gut microbiome (181); however, other evidence indicates that high-sugar diets can alter microbial composition (182, 183) and increase circulating LPS levels (184). LPS activates inflammatory pathways, particularly in the liver, through TLR4 signalling (185), which triggers NF-κB signalling pathway and promotes the production of pro-inflammatory cytokines such as TNF-α, IL-1β, and IL-6 (186) as well as generation of ROS (187). These events lead to hepatic inflammation and contribute to development of insulin resistance (188).

Gut microbiome dysbiosis in humans has been linked to the development and progression of cancer, primarily due to the carcinogenic effects of certain microbial metabolites and chronic inflammation resulting from host–microbiome interactions (189). In cancer, dysbiosis-driven signalling networks enhance pro-tumorigenic inflammation. Sustained PRR activation reinforces NF-κB activity and increases production of cytokines such as IL-6, IL-8, and TNF-α (190). Elevated IL-6 then activates the JAK/STAT3 pathway in both immune and epithelial cells, driving differentiation of naive CD4^+^ T cells into Th17 cells and enhancing secretion of IL-17A and IL-22 (191). IL-17A, in turn, promotes disruption of epithelial tight-junctions and recruitment of additional neutrophils, creating a self-reinforcing cycle of barrier dysfunction and inflammation (192, 193).

In contrast, metabolites produced by a balanced gut microbiota can have anticancer effects. SCFAs help regulate blood glucose levels (194) and may have cancer-preventive properties (195, 196). Other gut microbiota-derived compounds with anticancer potential include bacteriocins and phenylpropanoid derivatives, which have shown cytotoxic and anti-proliferative effects on various cancer cell lines (197–200), including bladder cancer cells (201).

Recent studies have demonstrated the role of gut microbiota in the development of bladder cancer and supported the concept of a functional gut-bladder axis (202–204) Yang et al. (204) identified five taxa of the gut microbiome associated with bladder cancer, with the genus *Bilophila* linked to an increased risk of bladder cancer. Interestingly, the genus *Bilophila* shows a positive correlation with diets high in animal protein, while it is negatively linked to the consumption of plant-based proteins (205). Using a mouse model of bladder cancer induced by the N-butyl-N-(4-hydroxybutyl)-nitrosamine (BBN), Roje et al. (206) found that depleting the gut microbiome markedly reduced bladder tumour development, demonstrating that gut bacteria bioactivate this carcinogen. Multiple bacterial strains in mice and humans were identified as capable of converting BBN into the genotoxic metabolite N-butyl-N-(3-carboxypropyl) nitrosamine (BCPN), which accumulates in the bladder and induces DNA damage. Analysis of human-derived microbiota revealed significant inter-individual variation in BBN-to-BCPN conversion, suggesting that gut microbial composition may influence risk of bladder cancer.

Current strategies to manage gut microbiome dysbiosis include faecal microbiota transplantation, genetically engineered bacteria, and more accessible methods like high-fibre diets, fermented foods, probiotics, and prebiotics (reviewed in (207)). Among these, probiotics are gaining recognition for supporting blood glucose control (208) and cancer prevention (209). Lactic acid bacteria strains, for instance, may enhance insulin sensitivity, improve gut barrier integrity, and reduce inflammation in diabetic or prediabetic individuals (210, 211). While most probiotic cancer therapies remain in preclinical stages, some clinical studies show promise. For example, *Lactobacillus casei* has demonstrated potential in reducing recurrence risk in non-muscle invasive bladder cancer, supporting its use as adjuvant therapy (212–214). Based on preclinical studies, it is hypothesized that *Lactobacillus casei* may enhance cell-mediated immunity by activating dendritic cells, boosting NK cell activity, and stimulating the production of anti-tumour cytokines such as IFN-γ and TNF-α (215, 216). Nevertheless, the exact immunological mechanisms in patients remain speculative.

In certain cases, particularly in the treatment of T2DM, some medications have been useful for lowering blood glucose levels. Metformin, the most studied medication among them, lowers blood glucose by activating the adenosine monophosphate-activated protein kinase (AMPK) pathway, enhancing muscle glucose uptake and reducing insulin levels (217). Epidemiologic studies suggest that metformin users have a lower risk of developing various types of cancer (218). Regarding bladder cancer, metformin may lower the risk of recurrence and cancer-specific mortality in muscle-invasive bladder cancer, though it shows limited impact on non-muscle-invasive cases (219, 220). While some studies found no clear overall benefit, metformin’s positive effects on recurrence appear more evident with longer follow-up periods (221). Besides regulation of blood glucose, metformin may have direct anti-cancer effects through mechanisms involving mitochondrial inhibition, AMPK activation, and pathways like IGF-1R/PI3K/AKT/mTOR and p53. These impact cancer hallmarks such as cell cycle control, apoptosis, and immune response, though the exact mechanisms remain unclear (reviewed in (222)). Metformin has also been found to alter the composition of the gut microbiota in diabetics (223), reflecting the effects of a plant-based diet by promoting the growth of beneficial and SCFA-producing bacteria (224). However, in healthy individuals, no significant changes in gut microbial diversity were observed following metformin treatment (225).

Several other medications are available for the management of blood glucose levels. One increasingly popular drug is semaglutide, a glucagon-like peptide-1 (GLP-1) receptor agonist. Although the data available to date is very sparse, there are indications that semaglutide, like metformin, could have a dual benefit: blood glucose control and potential cancer risk reduction (226) (Figure 3).

This article emphasizes diet as a key factor in maintaining healthy blood sugar levels and reducing systemic inflammation, yet evidence also highlights the significant role of regular physical activity. Regular exercise manages microbiome dysbiosis by increasing microbial diversity, boosting SCFAs metabolism, and promoting the proliferation of commensal bacterial populations (227, 228). Large-scale studies have found a strong inverse association between physical activity and various cancers, including bladder cancer (229, 230). Moreover, physical activity offers benefits in both cancer prevention and treatment support (reviewed in (231)), as it has been shown to significantly reduce the adverse effects of cancer therapies, supporting its role in routine cancer treatment protocols (232).

Ultimately, while medications can support metabolic health by maintaining glucose homeostasis and reducing systemic inflammation, they only address the symptoms and not the causes of the disease and are therefore less effective in the long term. Sustainable health still relies heavily on lifestyle choices, as what we eat and how active we are daily can be the deciding factor between chronic disease and health.

## 7 Conclusion

High intake of sugar and refined carbohydrates with a high glycaemic load remains common, particularly in Western diets, and significantly increases the risk of obesity and metabolic disorders by affecting body fat, insulin sensitivity, and glucose metabolism. The growing body of evidence highlights a link between sugar metabolism, chronic inflammation, and the onset and progression of bladder cancer. Both experimental and epidemiological studies indicate that hyperglycaemia and insulin resistance, primarily driven by high-sugar and high-glycaemic diets, contribute to microbiota imbalance, a pro-inflammatory environment, and oxidative stress, all of which are drivers of bladder carcinogenesis. Although some inconsistencies remain across studies, the cumulative findings underscore the need for a paradigm shift in bladder cancer prevention and management, prioritising dietary quality, metabolic health, and inflammation control as core components.

Further research is required to clarify causality and explore the therapeutic potential of targeting inflammation and metabolic pathways in bladder cancer. However, maintaining a diet low in sugar and refined carbohydrates and high in fibre supports metabolic health, reduces inflammation, and has been shown to be a practical and accessible strategy to lower bladder cancer risk and improve clinical outcomes.
